# Editorial: Transitions to Sustainable Food and Feed Systems

**DOI:** 10.3389/fpls.2019.01283

**Published:** 2019-10-18

**Authors:** Marta Wilton Vasconcelos, Bálint Balázs, Eszter Kelemen, Geoffrey R. Squire, Pietro P. M. Iannetta

**Affiliations:** ^1^CBQF—Centro de Biotecnologia e Química Fina–Laboratório Associado, Escola Superior de Biotecnologia, Universidade Católica Portuguesa, Porto, Portugal; ^2^Environmental Social Science Research Group, Budapest, Hungary; ^3^Institute of Sociology, Hungarian Academy of Sciences, Budapest, Hungary; ^4^The James Hutton Institute, Dundee, United Kingdom

**Keywords:** sustainable food, feed system, food system, policy making, agricultural practice

Fundamental changes in our current food systems must be tackled to ensure that human populations have access to foods and feeds that are produced, processed, and marketed in a manner which is biodiversity-sensitive and delivers environmental benefits. This is especially important since the “Anthropocene” is not simply characterized by geochemical cycles whose levels exceed planetary boundaries, but also by the rising costs to human health of over-consumption and resource inefficiencies. Thus, sustainable consumption demands foods and feeds which are proven to be highly nutritious, safe, and meet quality standards. Assuring such an aim entails overcoming barriers, or more positively, realizing opportunities. Sustainable food chains, or more correctly “food” and “feed value networks” can be realized by integrating “push” and “pull” approaches. Pushing of biodiverse- and environmentally sensitive agricultural practice is allied with the pulling approaches of consumers to achieve good health. Across all sectors, policies on trade and education and associated governance structures need to be suitably tailored to accommodate regional and national pedoclimatic, socioeconomic, cultural contexts.

Among these considerations are how to implement measures which mitigate and adapt to climatic shifts, to reduce the dominance of feed (not food) security and to increase the number of crops within the production environment as well as the manner in which they are cultivated and processed. Achieving truly sustainable food and feed value networks will depend on: 1) understanding the connections and incongruences of the components which make up the current food and feed systems, and from historical as well as modern-day perspectives; 2) realizing social- and governance-structures that enable all stakeholders to become properly informed regarding the state of the current systems and options to improve them; 3) implementing chosen management options to ensure that sustainable economic development goals are harmonized for society and the environment as a whole.

Whole system-level accounting of food and feed value networks has still to be achieved and toward that end, this Research Topic compiles original research and perspective articles that contribute the necessary background information which will help the array of stakeholders which make-up civil societies to co-design their own transition path toward sustainable food and feed value networks.

Pulses are increasingly portrayed as key to break out of the sociotechnical lock-in of current agri-food systems which promote monocultures and animal feedlots. Magrini et al. assess the prospects of a transition of the agri-food system toward more diversified agriculture and sustainable consumption and identify innovation paths for pulses both downstream and upstream of agri-food supply chains. Public policies and institutions should have a significant role in supporting such a transition to ensure the simultaneous evolution of the downstream and upstream of the agri-food value chain. In particular, the authors highlight the need for investment in new storage facilities and processing technologies; more-effective sharing of knowledge on innovative cropping systems; and policy-making to ensure education which will increase the consumption of locally produced pulses, and so fulfill their positive personal, environmental, and economic potential.

In a unique study, Ritchie et al. highlight that calorific approaches are an inadequate means by which to reduce malnutrition. Instead they applied a novel “field-to-fork” approach to give the current best-estimate of essential nutritional provision at a global scale. The authors conclude that field-to-fork components of food networks should be characterized more fully to identify key bottlenecks (and opportunities) where nutritional provisions may be safeguarded, since that potential should not be assumed as a simple function of productivity. Therefore, the manuscript reports a holistic or whole system-level approach to nutritional provision. The findings point toward the need for more and better data, and especially with respect to the characterization of food networks, including the scale and localization of capacities with respect to relative need. 

Despite the considerable benefits provided by grain legumes, their production is constant in many countries, including the World’s number one pulse consumer, India. Smith et al. identified socioeconomic and agronomic barriers of pulse production by valorizing the traditional technical knowledge of small-scale Indian farmers. The major barriers—water shortage, lack of knowledge and funds, poor soils, limited availability of seeds, processing facilities, fertilizers, and fencing material—were found to be interlinked and dependent on the broader agri-food system. While some immediate measures are offered to increase production in the short term, staple changes can only be expected if policy decisions are applied in a multisectoral approach.

Technical innovations in agriculture are rarely effective and may be damaging when applied in ignorance of the systems in which they are to be deployed, or in the absence of consensus between companies, farmers, retailers, and consumers. Lotz et al. consider innovations, primarily in the topic of pest management, by reference to four case studies. In that of weed control in maize, a combination of policy instruments, commercial incentives, and appropriate extension advice ensured integration of chemical and mechanical control. When policy was repealed, control moved largely to chemical, the result being an increased pesticide load on the environment. The authors illustrate the widespread benefits of integration along the supply chain through *inter alia* directed policy support, eco-labeling, and state-of-the-art decision support. 

Can one optimize diets for environmental impact while maintaining a high nutrient balance? Kramer et al. looked at the feasibility of such nutritionally and environmentally appropriate diets within the context of the current Dutch eating habits. In the optimized diet they compared bread, breakfast cereals, dairy, and meat to point out that increasing bread and breakfast cereals in the optimized diet had the lowest environmental impact, while maintaining their nutrient balance. In contrast, dairy and meat increased the environmental impact significantly, and even at a 0% content of an optimized diet, the nutrient balance was maintained. The authors conclude that de-animalization of our current food culture would lead to more balanced and healthier nutrient intake and environmental impact. 

In the article by Richie et al. the authors looked at the specific scenario of hidden hunger, and its prevalence in India. This manuscript proposes concrete measures for reducing the incidence of micronutrient malnutrition *via* short (urgent), near-term (2030), and long-term (2050) actions. Scenario analysis to 2030 and 2050 indicate that losses in the supply chain need to be reduced. Broad strategies include increased intake of meat and dairy, since meat intake is very low in India and could be increased with benefit to nutrition but without major impact on the environment. Authors also point out to the importance of increasing pulse and pulse-derived products. However, the authors point out the lack of market access to the latter in this region. Measures for increased crop yields and production as well as targeted interventions of fortification, biofortification, and supplementation will continue to play a major role.

Lysine is an essential amino acid in human diets for which both animal- and plant-based sources are relevant. Leinonen et al. provided an innovative study showing that provision of lysine from plant-based sources is mostly attributed to soybean and they emphasize the need to promote the diversification of plant- and especially pulse-based lysine sources, and processing capacities. In this study, authors also provide a new assessment of the current sources of proteins in the human diet and analyze the possibilities for increasing the use of plant-based sources. The results demonstrate the importance of studies that look not simply at total protein levels, but importantly at individual nutrient provision, and in this case amino acid complement and levels of the essential “amino acids” which animals must consume as they cannot be synthesized metabolically. They also demonstrate that replacing animal protein and soya with other lysine-rich protein crops will come with obstacles, since major deviations in the assembly of global food systems will be needed.

## Final Comments

The papers presented in this topic highlight the need for a reappraisal of commonly used terms such as “food security” or “food chain.” First, food and feed supply are not usually the function of a simple “chain,” but rather several interconnected chains or “networks.” Furthermore, these networks do not simply deliver food, they also deliver feed and a range of industrial products including fibers, oils, and biomass for energy. So, production systems which are often stereotyped as food networks produce a diversity of outputs that influence many aspects of environmental, societal, and economic status of society. It is these impacts, and particularly the negative impacts, that have come to determine a shift in society’s “values” with respect to agriculture and food. That is, these values determine the impacts society will tolerate or wish to achieve, and expose what society really considers to be most beneficial and important. There is good cause therefore that the scientific community should act accordingly and opt to use the best-fit-for-purpose terms, and in the future include and elaborate upon the wider values desired in food and feed networks. Respecting the diversity of values among various stakeholders will be key to progressing dialogues, and especially those targeted toward more effective policies, with greater potential to help harmonize currently dominant values, such as those driven by largely economic considerations, with those which are neglected, such as societal and environmental wellbeing. Toward that end, an overarching question for those seeking more sustainable consumption might be: *what are our values, and how can we best assess that our food and feed networks are achieving these aspirations?*

## Author Contributions

All authors listed have made a substantial, direct, and intellectual contribution to the work, and approved it for publication.

## Funding

All the Editors of the Special Edition are supported by the European Union’s Horizon-2020 Research and Innovation project, “TRansition paths to sUstainable legume based systems in Europe,” www.true-project.eu under Grant Agreement Number 727973. PPMI is also supported by Rural and Environment Science and Analytical Services (RESAS), a division of the Scottish Government. MV is also supported by National Funds from FCT—Fundação para a Ciência e a Tecnologia through project UID/Multi/50016/2019. EK is also supported by János Bolyai Research Scholarship of the Hungarian Academy of Sciences.

## Conflict of Interest

The authors declare that the research was conducted in the absence of any commercial or financial relationships that could be construed as a potential conflict of interest.

